# EGF-Dependent Activation of ELK1 Contributes to the Induction of CLDND1 Expression Involved in Tight Junction Formation

**DOI:** 10.3390/biomedicines10081792

**Published:** 2022-07-26

**Authors:** Hiroshi Matsuoka, Alice Yamaoka, Takahiro Hamashima, Akiho Shima, Marin Kosako, Yuma Tahara, Jun Kamishikiryo, Akihiro Michihara

**Affiliations:** 1 Laboratory of Genomic Function and Pathophysiology, Faculty of Pharmacy and Pharmaceutical Sciences, Fukuyama University, Fukuyama, Hiroshima 729-0292, Japan; p7117131@fukuyama-u.ac.jp (A.Y.); smile_piero.taka0105@ezweb.ne.jp (T.H.); p7118040@fukuyama-u.ac.jp (M.K.); p7116086@fukuyama-u.ac.jp (Y.T.); 2 Department of Biochemistry, Faculty of Pharmacy and Pharmaceutical Sciences, Fukuyama University, Fukuyama, Hiroshima 729-0292, Japan; a-shima@fukuyama-u.ac.jp (A.S.); j-kamishi@fukuyama-u.ac.jp (J.K.)

**Keywords:** claudin domain-containing 1, gefitinib, epidermal growth factor receptor, ETS domain-containing protein-1, tight junction, anticancer

## Abstract

Claudin proteins are intercellular adhesion molecules. Increased claudin domain-containing 1 (CLDND1) expression is associated with the malignant transformation of estrogen receptor-negative breast cancer cells with low sensitivity to hormone therapy. Abnormal CLDND1 expression is also implicated in vascular diseases. Previously, we investigated the regulatory mechanism underlying CLDND1 expression and identified a strong enhancer region near the promoter. In silico analysis of the sequence showed high homology to the ETS domain-containing protein-1 (ELK1)-binding sequence which is involved in cell growth, differentiation, angiogenesis, and cancer. Transcriptional ELK1 activation is associated with the mitogen-activated protein kinase (MAPK) signaling cascade originating from the epidermal growth factor receptor (EGFR). Here, we evaluated the effect of gefitinib, an EGFR tyrosine kinase inhibitor, on the suppression of CLDND1 expression using ELK1 overexpression in luciferase reporter and chromatin immunoprecipitation assays. ELK1 was found to be an activator of the enhancer region, and its transient expression increased that of CLDND1 at the mRNA and protein levels. CLDND1 expression was increased following EGF-induced ELK1 phosphorylation. Furthermore, this increase in CLDND1 was significantly suppressed by gefitinib. Therefore, EGF-dependent activation of ELK1 contributes to the induction of CLDND1 expression. These findings open avenues for the development of new anticancer agents targeting CLDND1.

## 1. Introduction

The claudin (CLDN) family of proteins is a major component of tight junctions (TJs) in epithelial cells and the vascular endothelial cells that comprise epithelial tissues [1]. They are four-fold transmembrane proteins with two extracellular loops, and their amino- and carboxy-terminal tails extend into the cytoplasm and polymerize linearly to form TJ strands that regulate the permeability of the intercellular barrier [2]. These proteins play an important role in permeability, cell proliferation, and signal transduction [3,4]. In humans, 27 types of claudins have been identified, with a varying distribution across tissues based on the cell type and disease [5]. Aberrant expression of claudins has been reported in cancer tissues. For example, CLDN3 and CLDN4 are overexpressed in ovarian and breast cancers [6,7], whereas CLDN6 is overexpressed in germ cell tumors, gastric adenocarcinoma, lung adenocarcinoma, uterine cancer, and ovarian cancer [8]. Thus, the aberrant expression of claudin in a variety of cancers suggests that it plays a specific role in tumorigenesis. Notably, unregulated claudin plays an oncogenic role in target tissues and cell types.

Anti-CLDN4 antibodies have been reported to increase antitumor efficacy when used in combination with chemotherapeutic agents in colorectal, bladder, breast, and gastric cancers [9,10,11,12]. In addition, the anti-CLDN18.2 antibody (zolvecoximab/IMAB362) is more effective in the treatment of patients with advanced gastric/gastroesophageal junction and esophageal adenocarcinoma with positive CLDN18.2 expression than epirubicin, oxaliplatin, and capecitabine combination (EOX). Moreover, EOX in combination with zolvecoximab reportedly results in longer progression-free survival (PFS) and overall survival (OS) than EOX alone [13]. Claudin expression has also been shown to be related to cell death resistance in some tumors [14]. This suggests that targeting CLDNs might be a strategic approach in cancer therapy. Furthermore, claudin expression profiles are associated with patient prognosis in several cancer types. Understanding the claudin expression patterns in various disease states may lead to the use of claudin as an essential biomarker for cancer diagnosis.

Global expression analysis of basal-like breast cancer cell lines following the induction of apoptosis has suggested a link between claudin domain-containing 1 (CLDND1) and apoptosis [15]. CLDND1, also referred to as claudin 25, is highly homologous to other claudins and is localized in the TJs and cytoplasm [16]. Knockdown experiments suppressing CLDND1 expression have demonstrated nuclear fragmentation, cleavage of caspase-3, and release of cytochrome C from the mitochondria, indicating the induction of apoptosis. Furthermore, inhibition of kinase signaling by the MEK1/2-ERK1/2 and JNK pathways has been shown to markedly enhance apoptosis by CLDND1 knockdown. Thus, the targeted regulation of CLDND1 and the kinase signaling pathway may lead to the development of novel therapies for cancer [15].

In our previous study, we showed that CLDND1 expression was decreased at the protein level in the hemorrhage site of mice with collagenase-induced cerebral hemorrhage, and knockdown culture cell experiments revealed that the decrease in CLDND1 expression increased the permeability of the intercellular barrier [17]. In addition, CLDND1 is expressed in the liver, testes, and brain, with the expression distribution strongly correlating with retinoic acid receptor-related orphan receptor α (RORα), which is involved in the transcriptional regulation of CLDND1 [18,19]. RORα relies on cholesterol as a ligand to regulate transcription [20,21], and inhibition of cholesterol synthesis by statin treatment has been shown to reduce the transcriptional regulation of CLDND1 by RORα [22]. The post-transcriptional regulation of CLDND1 is regulated by microRNA-124 [23]. Furthermore, in an attempt to elucidate novel mechanisms involved in the regulation of CLDND1 expression, we identified an enhancer of CLDND1 expression and a transcription factor, myeloid zinc finger 1 (MZF1), which is bound to the enhancer of the CLDND1 promoter region, using the luciferase reporter assay with stepwise deletion and chromatin immunoprecipitation analysis [24,25]. It has been reported that gene expression is regulated by the interaction of multiple transcription factors [26]. Consequently, we searched for transcription factors that could bind to this enhancer and found a high homology to the ETS domain-containing protein-1 (ELK1) binding sequence.

ELK1 functions as a transcription factor and is a member of the ternary complex factor (TCF) subgroup of the E twenty-six (Ets) oncogene family, which is involved in various processes, including cell growth and differentiation, angiogenesis, and cancer [27]. The transcriptional activity of ELK1 is induced by the phosphorylation of MEK1/2-ERK1/2, JNK, and p38, a MAPK signaling cascade mediated by the stimulation of EGFR [28,29,30,31]. ELK1 phosphorylation has been shown to promote binding to serum response elements (SREs) by forming a complex with the serum response factor (SRF) [32]. The MEK1/2-ERK1/2 pathway is associated with the growth of various cancer cells, including breast cancer, hepatocellular carcinoma, and lung cancer [33,34,35]. Gefitinib (Iressa, ZD1839), an orally available low molecular weight quinazoline derivative, is an EGFR tyrosine kinase inhibitor (EGFR-TKI) that selectively inhibits the MEK1/2-ERK1/2 cascade by blocking EGFR autophosphorylation and inducing apoptosis of EGF-stimulated tumor cells [36]. This targeted molecular drug plays an important role in clinical practice by significantly prolonging the median survival of patients with non-small cell lung cancer [37].

Therefore, we hypothesized that ELK1 phosphorylation by the EGF-stimulated MEK1/2-ERK1/2 pathway regulates the transcription of CLDND1. To test this hypothesis, we used a vascular endothelial cell culture system in which CLDND1 is highly expressed to elucidate the mechanism by which CLDND1 expression is regulated by EGF-dependent activation of ELK1 and to evaluate the effect of gefitinib treatment on the regulation of CLDND1 expression.

## 2. Materials and Methods

### 2.1. Cell Culture

Immortalized human brain endothelial cells (HBECs) were obtained from the American Type Culture Collection (Manassas, VA, USA). The cells were grown in Dulbecco’s modified Eagle’s medium (DMEM) (FUJIFILM Wako Pure Chemical Co., Osaka, Japan) supplemented with 100 μg/mL penicillin–streptomycin solution (Thermo Fisher Scientific, Waltham, MA, USA) and 10% fetal bovine serum (FBS) (Biological Industries, Beit HaEmek, Israel) at 37 °C in a 5% CO_2_ atmosphere.

### 2.2. Luciferase Reporter and Expression Constructs

For the construction of the luciferase reporter, a DNA fragment containing the human CLDND1 promoter region (–742 to +192, or –734 to +192, with the transcription start point at +1) was first amplified using polymerase chain reaction (PCR) with the genomic DNA of HBECs. The DNA fragment was treated with restriction enzymes, MluI and SalI, and cloned into the MluI/XhoI site of the luciferase expression vector pGVB2. To construct the expression vector, the DNA fragment of the ORF region of ELK1 wild-type, ELK1-del lacking the DNA-binding domain in the N terminal up to the 86th residue [38], ELK1-S383A with alanine substitution in the phosphorylation site of serine 383 residue [39], or SRF was first amplified using PCR with cDNA synthesized via reverse transcription from mRNA isolated after culturing HBECs. The DNA fragments were cloned into the EcoRV/XhoI site of the expression vector containing the CMV promoter pcDNA3.1-Flag-HA (Nippon Gene, Tokyo, Japan), after restriction treatment with EcoRV/XhoI. The cloned vector DNAs were purified using the Qiagen Plasmid Mini Kit (Qiagen, Hilden, Germany), and the sequences were confirmed via sequencing. The primers used are listed in Supplementary Table S1.

### 2.3. Transfection and Luciferase Activity Assay

Transfection was performed according to a previously described method [18]. Before the day of transfection, the cells were seeded in 24-well plates containing DMEM with 10% FBS at a density of 0.5 × 10⁵ cells/well. Each well was seeded with a mixture of 100 ng of ELK1- or ELK1 mutant-expressing vector, 200 ng of luciferase reporter vector, and 200 ng of β-galactosidase reporter vector, transfected using Lipofectamine 2000 (Thermo Fisher Scientific, Waltham, MA, USA) for 15 h. The cells were cultured for 30 h in fresh medium and lysed. Luciferase activity was measured using the PicaGene kit (Toyo Ink Co., Ltd., Tokyo, Japan) according to the manufacturer’s instructions and Luminescencer-PSN AB-2200 (Atto Co., Ltd., Tokyo, Japan).

### 2.4. Chromatin Immunoprecipitation (ChIP) Assay

HBECs were seeded at densities of 4 × 10⁵ cells/well in six-well plates containing DMEM supplemented with 10% FBS. The cells were cultured until a confluence of 80–90% at 37 °C in a 5% CO_2_ atmosphere, and then transfected for 15 h with a mixture of 500 ng of luciferase reporter vector (–1017/+192 or –734/+192) and 500 ng of ELK1- (wild-type or mutant-type) or SRF-expressing vector. Next, the cells were cultured for 72 h in fresh medium, and then treated with 100 ng/mL EGF for 1 h. The ChIP assay was conducted using the OneDay ChIP kit (Diagenode, Liège, Belgium), according to the manufacturer’s instructions. The reaction mixtures were incubated overnight at 4 °C, with the addition of 1 μg of mouse anti-FLAG antibody (Merck Millipore, Billerica, MA, USA), mouse anti-ELK1 antibody (E-5, Santa Cruz Biotechnology, Santa Cruz, CA, USA), or non-immunized IgG as a negative control. Antibody-bounded protein-DNA complexes were concentrated by incubation with unblocked protein A beads (Diagenode) for 1 h at 4°C, and the bound DNA was purified according to the manufacturer’s instructions. The bound DNA was detected using PCR, and the relative binding intensity was calculated using quantitative PCR with SYBR Green Real-Time PCR Master Mix (Toyobo, Osaka, Japan) in a LightCycler real-time PCR system (Roche, Penzberg, Germany). The primers used are listed in Supplementary Table S1.

### 2.5. Reverse Transcription-Quantitative PCR (RT-qPCR)

Transfection was performed according to a previously described procedure [18]. HBECs were seeded in 12-well plates at a density of 2 × 10⁵ cells/well and transfected with 500 ng of empty or ELK1-expressing vector for 15 h. The cells were then cultured for 72 h in fresh medium and lysed. For EGF treatment, the cells were spread and grown to approximately 80–90% confluency, and then treated with 10 ng/mL EGF for 30, 60, or 90 min, and 1, 10, or 100 ng/mL EGF for 60 min. The cells were treated with 1 μmol/L gefitinib for 60 min, followed by treatment with 100 ng/mL EGF for 60 min. The total RNA was prepared using ISOGEN (Nippon Gene, Co., Ltd., Tokyo, Japan) according to the manufacturer’s protocol and reverse-transcribed for 90 min at 37 °C using 200 U Moloney murine leukemia virus reverse transcriptase (Thermo Fisher Scientific, Waltham, MA, USA). The cDNA was amplified using a LightCycler real-time PCR system (Roche, Penzberg, Germany); 12-μL reaction mixture contained SYBR Green Real-Time PCR Master Mix (Toyobo, Osaka, Japan) and 1 μM gene-specific primers. The primers used are listed in Supplementary Table S1.

### 2.6. Western Blotting

Transfection was performed according to a previously described method [18]. Briefly, HBECs were seeded at densities of 4 × 10⁵ cells/well in six-well plates containing DMEM and transfected for 15 h with 500 ng of empty or ELK1-expressing vector. The cells were then grown for another 72 h in fresh medium and lysed in ice-cold lysis buffer containing 50 mM Tris-HCl, 200 mM sucrose, 1 mM ethylenediaminetetraacetic acid, 0.5 mM phenylmethylsulfonyl fluoride, 1 µg/mL pepstatin, 1 µg/mL leupeptin, and 1% sodium dodecyl sulfate (SDS). Equal amounts of protein extracts were resolved using SDS-polyacrylamide gel electrophoresis and electrotransferred onto Immobilon-P membranes (Merck Millipore). Protein detection was performed using mouse anti-FLAG antibody (Merck Millipore) and rabbit anti-CLDND1 antibody (custom antibody against the epitope DEADEKTYNDALFRYN produced by Eurofins Genomics [13054V; Tokyo, Japan]). Analyses were performed according to standard procedures as previously described [17].

### 2.7. Statistical Analysis

All experiments were performed at least three times. Data are expressed as mean ± standard error (SE) unless otherwise specified. Comparisons between two groups were performed using Student’s *t*-test. Values were considered statistically significant at *p* < 0.05. Comparisons among three or more groups were performed using a one-way analysis of variance (ANOVA).

## 3. Results

### 3.1. Effect of ELK1 on the Enhancer Region of CLDND1

**Figure 1:**
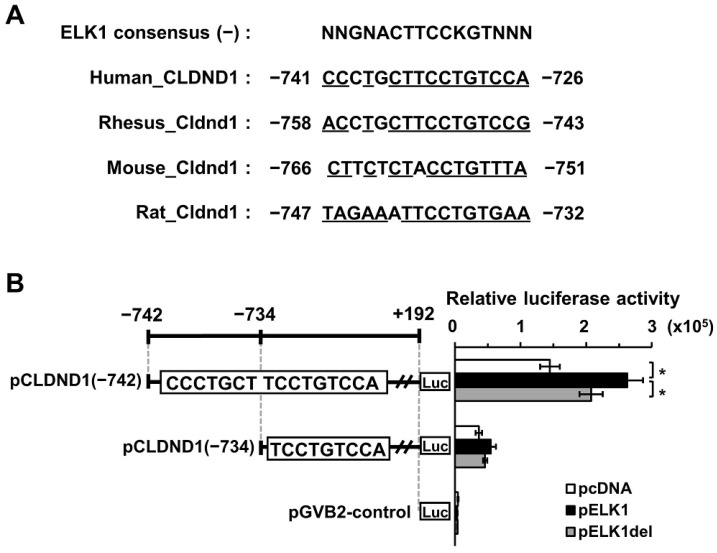
ELK1-binding sites in the CLDND1 promoter region. (**A**) Sequences of putative ELK1-binding sites in humans, rhesus monkeys, mice, and rats. Common sequences that are conserved among species are underlined. (**B**) HBECs were co-transfected with luciferase reporter vectors linked to the fragment regions –742 to +192 (pCLDND1(–742), with an enhancer) or –734 to +192 (pCLDND1(–734), without an enhancer), and empty (pcDNA), or ELK1-expressing (pELK1) or ELK1 deletion mutant-expressing (pELK1del) vectors, respectively. Results are expressed as mean ± SE, *n* = 3, * *p* < 0.05.

Through in silico analysis using TFBIND software [40], we searched for transcription factors that could bind to the enhancer region (–742 to –734) and found that they were highly homologous to ELK1-binding sequences. In addition, a comparison of the ELK1 consensus sequences from humans, rhesus monkeys, mice, and rats showed high homology to each other (Figure 1A).

To evaluate the responsiveness of ELK1 to enhancers, HBECs were used to co-transfect each fragment of –742 to +192 (with an enhancer) or –734 to +192 (without an enhancer) luciferase reporter with an empty or ELK1 (wild-type or mutant-type)-expressing vector, respectively, and luciferase activity was measured. The responsiveness of the enhancer-containing reporter showed a 1.8-fold increase in ELK1 overexpression compared with that of the empty vector. In contrast, the reporter without the enhancer did not show any change in activity. Furthermore, a comparison of ELK1 and ELK1del, which lacks the DNA-binding domain, revealed that ELK1del showed reduced responsiveness to the enhancer. Therefore, we proposed that ELK1 responded to the enhancer region as an activator (Figure 1B).

### 3.2. Binding of ELK1 and SRF to the Enhancer Region of CLDND1

**Figure 2:**
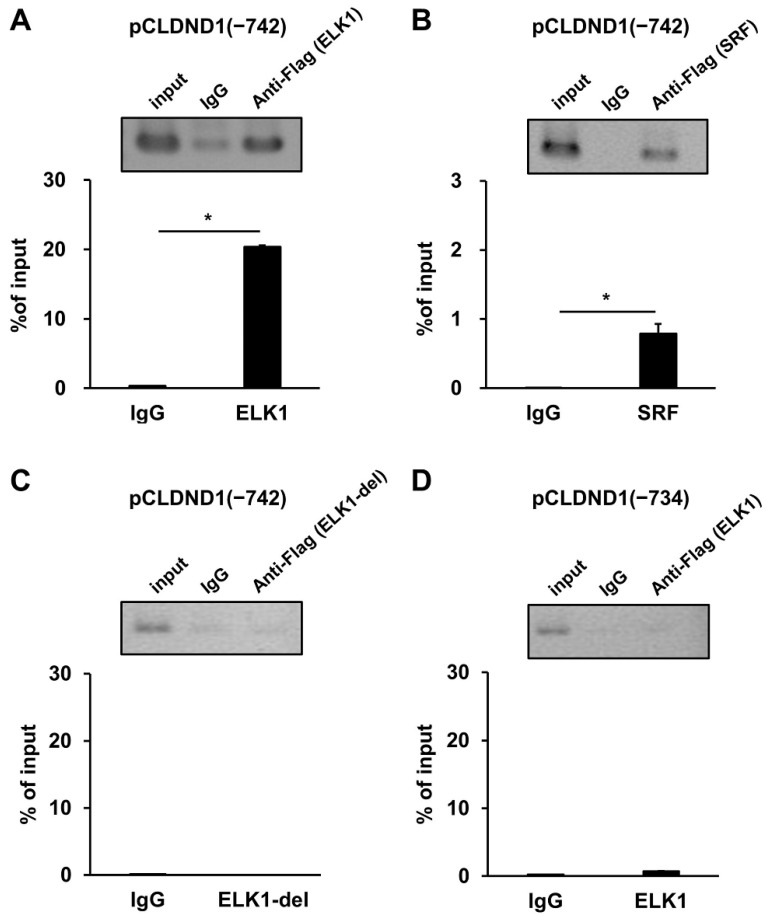
Binding of ELK1 and SRF to the enhancer of the CLDND1 promoter region. HBECs were co-transfected with a luciferase reporter vector containing the enhancer of the CLDND1 promoter region and ELK1-, SRF-, or ELK1 deletion (ELK1del) mutant-expressing vectors, and then the binding to ELK1 (**A**), SRF (**B**), or ELK1del (**C**) was measured. HBECs were co-transfected with a luciferase reporter vector lacking the enhancer of the CLDND1 promoter region and ELK1-expressing vectors, and then binding to ELK1 (**D**) was measured. ChIP analysis was performed using PCR product electrophoresis images (upper panels) and quantitative PCR (lower graphs). Results are expressed as mean ± SE, *n* = 5, * *p* < 0.05.

To evaluate the binding of ELK1 and SRF to the enhancer region of CLDND1, we performed ChIP-PCR using HBECs co-transfected with ELK1 or SRF transcription factors and the enhancer region of CLDND1. Immunoprecipitation of ELK1 or SRF with the anti-FLAG antibody showed approximately 66- or 150-fold higher binding than with the anti-IgG antibody, respectively (Figure 2A,B). The ChIP analysis with ELK1 mutations lacking the DNA binding domain also showed no binding to the enhancer region (Figure 2C). Furthermore, the ChIP analysis with a reporter of ELK1 expression and enhancer region deletion showed that ELK1 did not bind (Figure 2D).

### 3.3. Effect of ELK1 Overexpression on CLDND1 Expression

**Figure 3:**
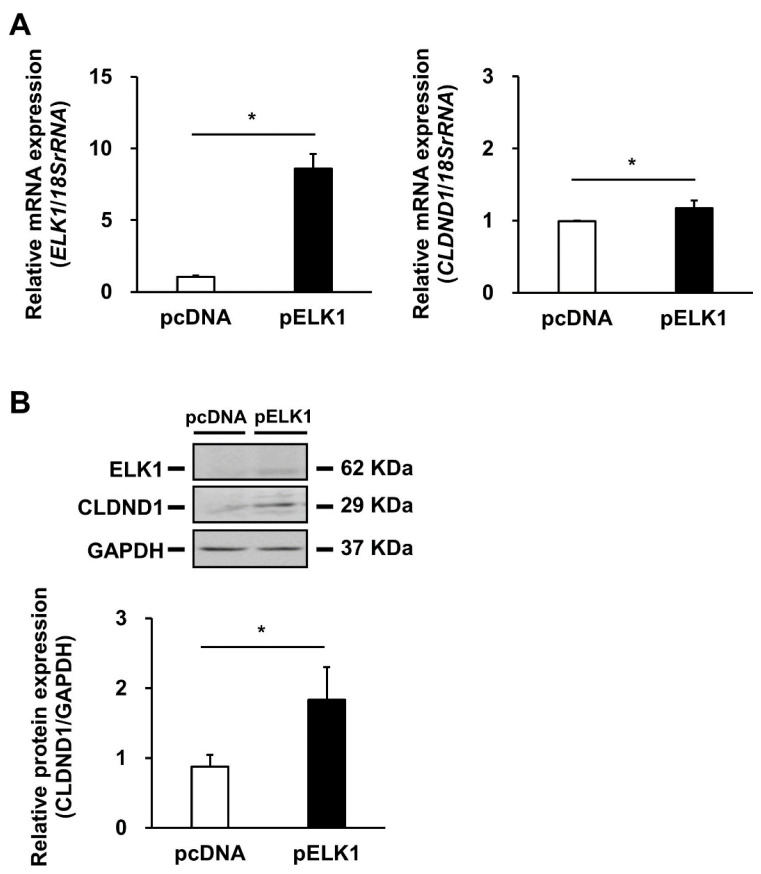
Effect of ELK1 overexpression on CLDND1 expression levels. (**A**) HBECs were transfected with an empty (pcDNA) or ELK1-expressing (pELK1) vector and incubated for 72 h. mRNA levels of ELK1 and CLDND1 were determined using qRT-PCR. 18S rRNA was used for internal correction. (**B**) HBECs were transfected with the pcDNA or pELK1 vector and incubated for 72 h. The protein levels of ELK1 and CLDND1 were determined using western blotting with the anti-FLAG and anti-CLDND1 antibodies, respectively. GAPDH was used for internal correction. Results are expressed as means ± SE, *n* = 3, * *p* < 0.05.

To quantify the mRNA expression levels of *CLDND1* following ELK1 overexpression, qRT-PCR was performed using HBECs transfected with an empty or ELK1-expressing vector. The results showed that the expression of *CLDND1* mRNA increased significantly by approximately 1.2-fold with an increase in ELK1 expression (Figure 3A). Similarly, western blotting was performed to quantify the expression levels of CLDND1 protein following ELK1 overexpression. The results showed that the expression of CLDND1 protein increased significantly by approximately two-fold with an increase in ELK1 expression (Figure 3B).

### 3.4. Effect of EGF Stimulation on CLDND1 Expression

**Figure 4:**
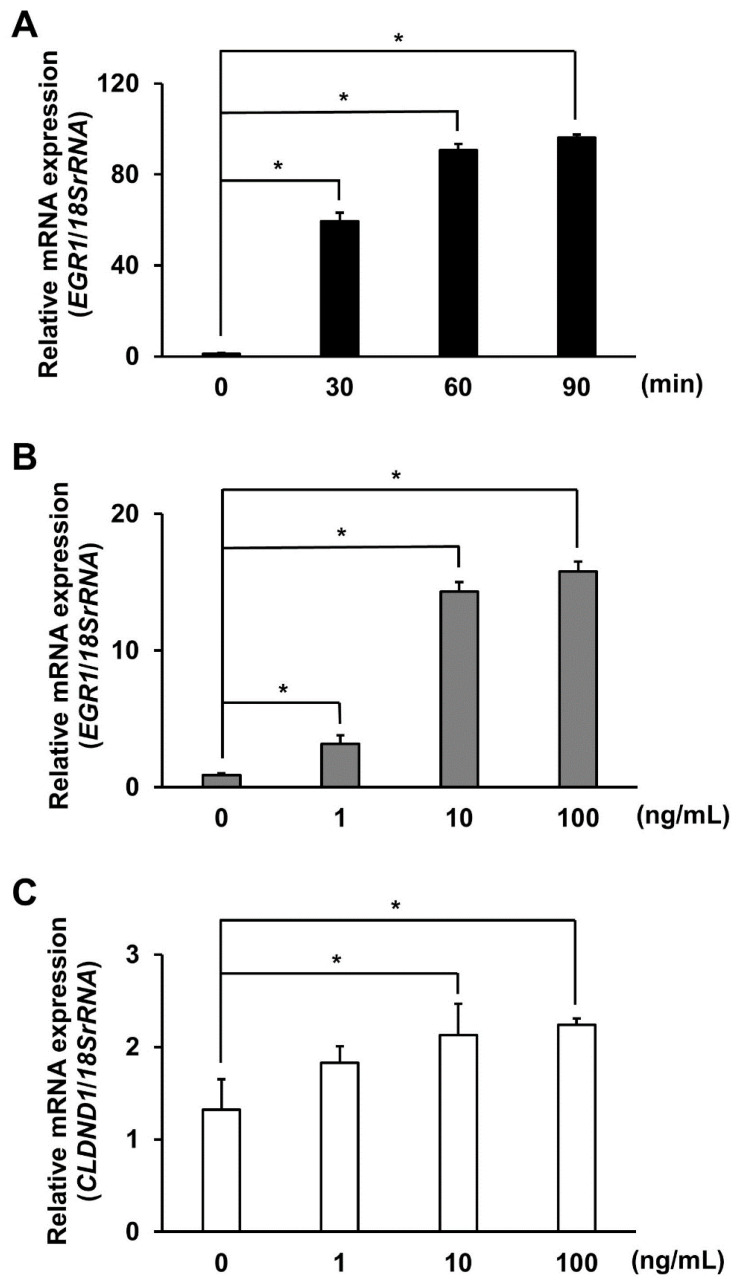
Effect of EGF treatment on CLDND1 expression. (**A**) HBECs were cultured in the presence of 0.1% FBS and treated with 10 ng/mL EGF for 0, 30, 60, or 90 min. mRNA levels of *EGR1* were measured using qRT-PCR, which was increased by EGF treatment as a positive control. (**B**) HBECs were cultured in the presence of 0.1% FBS and treated with 0, 1, 10, or 100 ng/mL EGF for 60 min. mRNA levels of *EGR1* were measured using qRT-PCR. (**C**) HBECs were cultured in the presence of 0.1% FBS and treated with 0, 1, 10, or 100 ng/mL EGF for 60 min. mRNA levels of *CLDND1* were measured using qRT-PCR. 18S rRNA was used for internal correction. Results are expressed as means ± SE, *n* = 3, * *p* < 0.05.

To evaluate EGF-stimulated ELK1 phosphorylation-mediated regulation of EGR1 mRNA expression, the expression levels of *EGR1* mRNA, which were increased by EGF treatment as a positive control, were evaluated using qRT-PCR. EGF (10 ng/mL) was added at 0, 30, 60, and 90 min. As a result, the expression levels of *EGR1* mRNA increased by approximately 100-fold as determined at 60 min, with the effect lasting up to 90 min (Figure 4A). In addition, treatment with EGF at concentrations of 1, 10, and 100 ng/mL for 60 min resulted in 3.5-, 16.1-, and 17.8-fold increases in the mRNA levels compared with that in untreated cells, respectively (Figure 4B). Similarly, to evaluate the expression levels of *CLDND1* mRNA following EGF stimulation, EGF was applied at concentrations of 0, 1, 10, and 100 ng/mL for 60 min, which resulted in 1.6- and 1.7-fold increases in the mRNA level compared with that in untreated cells at concentrations of 10 and 100 ng/mL, respectively (Figure 4C).

### 3.5. Effect of EGF Stimulation on the Binding of the ELK1 Complex to the CLDND1 Enhancer Region

**Figure 5:**
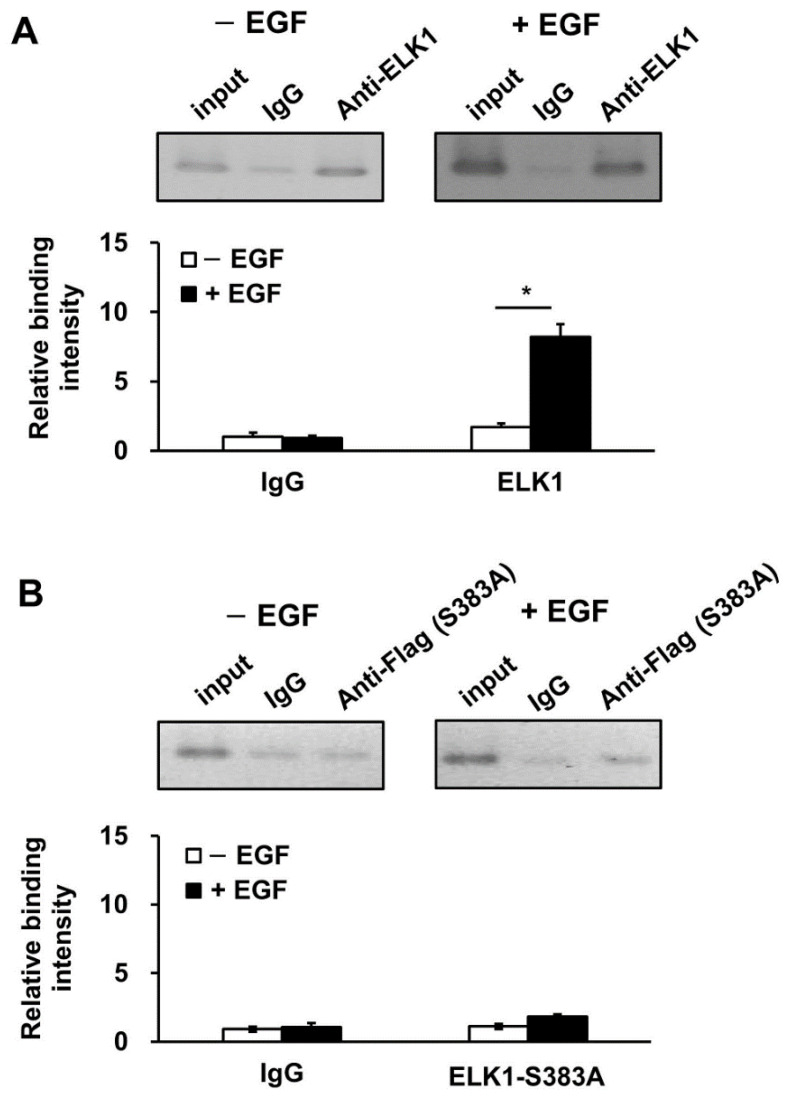
Effect of EGF treatment on DNA binding by ELK1 phosphorylation. HBECs were transfected with a luciferase reporter vector containing an enhancer region and ELK1 wild-type (**A**) or ELK1-S383A mutant-type (**B**), and SRF-expressing vectors and then treated with or without 100 ng/mL EGF for 60 min. ChIP-PCR was used to determine the binding ability of the ELK1-DNA complex using the anti-ELK1 antibody (**A**) or anti-Flag antibody (**B**) with PCR product electrophoresis images (upper panels). ChIP-PCR was performed using the non-immune IgG antibody as a control. The binding intensity by non-immune IgG and anti-ELK1 was calculated based on the band signals of ChIP-PCR using cells untreated and treated with EGF (lower graphs). Results are expressed as mean ± SE, *n* = 3, * *p* < 0.05.

To evaluate the binding of the ELK1 complex to the CLDND1 enhancer region following EGF treatment, HBECs were co-transfected with a reporter vector containing the enhancer region of CLDND1 and expression vectors of ELK1 and SRF. After 72 h, the cells were treated with 100 ng/mL EGF for 60 min. The binding of ELK1 to the enhancer was evaluated using ChIP-PCR. The results revealed that immunoprecipitation with the anti-ELK1 antibody in the EGF-treated and untreated groups showed an increase in binding in both groups compared with that observed for each band of the anti-IgG antibody (Figure 5A, upper panels). Next, the integrated values for each band were calculated from the imaging data. Immunoprecipitation with the anti-ELK1 antibody after EGF treatment showed approximately five-fold higher binding ability than that of the untreated EGF group (Figure 5A, lower graphs). To evaluate whether binding to the enhancer region is mediated by phosphorylated ELK1, we evaluated the effect of EGF treatment using non-phosphorylated ELK1 (ELK1-S383A), in which the phosphorylation site, the serine 383 residue, is substituted with alanine. The results showed that EGF treatment attenuated ELK1 binding to the enhancer region (Figure 5B).

### 3.6. Effect of Gefitinib Treatment on CLDND1 Expression Induced by EGF Stimulation

**Figure 6:**
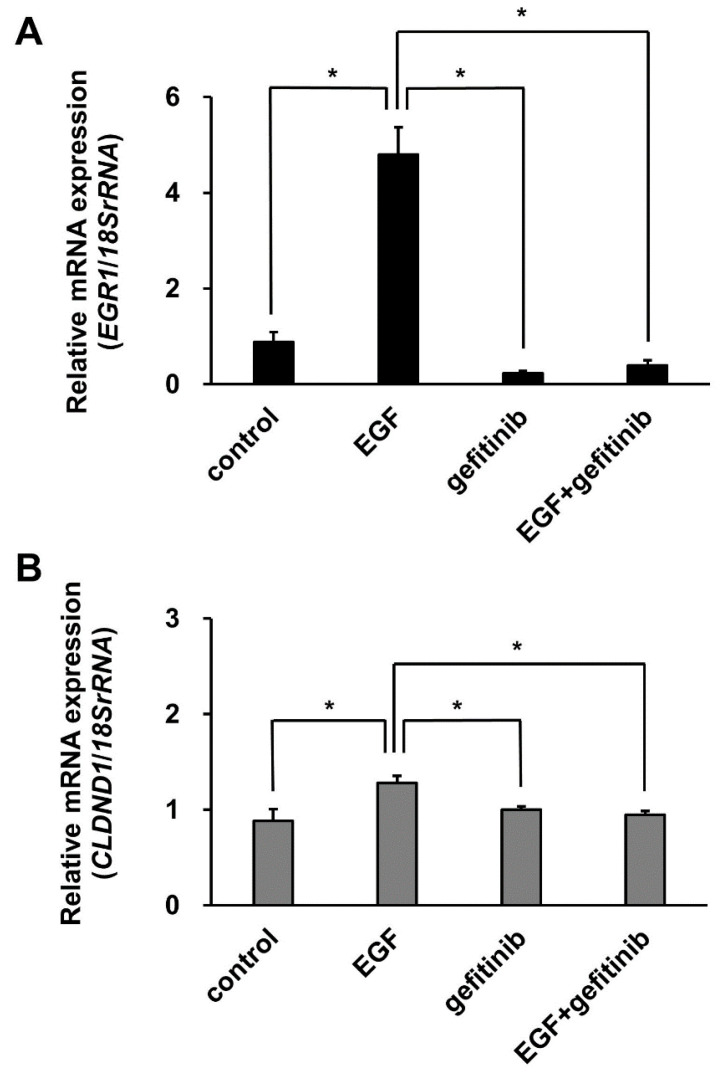
Effect of gefitinib treatment on CLDND1 expression. (**A**) HBECs were untreated or treated with 1 µmol/L gefitinib for 60 min and then untreated or treated with 100 ng/mL EGF, and total RNA was isolated after 60 min. The mRNA levels of *EGR1* were then measured using qRT-PCR. Results are expressed as mean ± SE, *n* = 5, * *p* < 0.05. (**B**) mRNA levels of *CLDND1* were measured using qRT-PCR. Results are expressed as mean ± SE, *n* = 3, * *p* < 0.05.

To evaluate the inhibitory effect of gefitinib on EGFR tyrosine kinase, the expression levels of *EGR1* mRNA induced by EGF stimulation were determined as a positive control using qRT-PCR in the non-treated, EGF-treated, gefitinib-treated, and gefitinib-EGF-treated groups. The results showed that the EGF-induced increase in EGR1 expression was significantly suppressed by approximately 90% in the gefitinib-EGF-treated group (Figure 6A). Similarly, to evaluate the expression levels of *CLDND1* mRNA following the addition of gefitinib, we performed qRT-PCR. The results showed that the EGF-induced increase in *CLDND1* expression was significantly suppressed by approximately 30% in the gefitinib-EGF-treated group (Figure 6B).

## 4. Discussion

In this study, we analyzed the regulatory mechanism of CLDND1 expression, which is involved in cancer cell proliferation and TJ formation. We found that gefitinib attenuated the upregulation of CLDND1 expression by EGF-mediated ELK1 phosphorylation.

In our previous study, we identified an enhancer at positions –742 to –734 using the luciferase reporter analysis with stepwise deletion of the CLDND1 promoter region [21]. In silico analysis revealed that MZF1 and ELK1 are transcription factors that bind to this enhancer. We found that MZF1 functions as an activator of CLDND1 expression and showed that *MZF1* knockdown cells exhibit increased intercellular permeability. This indicates that the suppression of MZF1 expression may decrease CLDND1 expression and cause defects in the formation of TJs [21].

ELK1, a member of the TCF subgroup of the Ets oncogene family, is involved in various processes, including cell growth and differentiation, angiogenesis, and cancer, and functions as a transcription factor [27]. The ELK1 consensus sequence within the enhancer showed a high homology in humans, rhesus monkeys, mice, and rats (Figure 1A). The luciferase reporter and ChIP assays suggested that ELK1 binding to the enhancer promotes transcription of CLDND1 (Figure 1B and Figure 2). In addition, ELK1 has been shown to bind to serum response elements (SREs) by forming a complex with SRF [32]. However, even though the enhancer we identified did not have SRE features, it showed SRF-binding properties (Figure 2). This suggests that SRF can bind to the ELK1-binding site. Furthermore, some members of the ELK family recognize and bind to the same binding sequence, which may also be regulated by other members of the ELK family [41]. We showed that ELK1 overexpression increased the expression levels of CLDND1 mRNA and protein (Figure 3A,B). This suggests that transcription of CLDND1 is dependent on ELK1 expression levels. ELK1 is activated through phosphorylation by MEK1/2-ERK1/2, JNK, and p38, a MAPK signaling cascade mediated by the stimulation of EGF [28,42]. Therefore, we evaluated the changes in CLDND1 expression upon EGF stimulation and found that CLDND1 expression was increased along with increased expression of EGR1 (Figure 4A–C), which has been reported to be responsive to EGF stimulation [43]. This suggests that EGF stimulation activates the MAPK pathway and increases the expression of CLDND1.

Moreover, we compared the binding ability of the ELK1 complex with and without EGF stimulation and found that the binding was stronger following EGF treatment (Figure 5A,B). This suggests that the activation of ELK1 phosphorylation enhances the binding ability of the enhancer. The addition of gefitinib, an EGFR-TKI used in clinical practice for diseases such as non-small cell lung cancer, significantly decreased the expression of CLDND1, which was increased by EGF (Figure 6B). Thus, the expression of CLDND1 decreased in accordance with the inhibition of the MAPK pathway by gefitinib. Gefitinib induces apoptosis by inhibiting EGFR and exerts anti-cancer effects by inhibiting angiogenesis through suppression of vascular endothelial growth factor production [36,44]. Part of the effect of gefitinib is its suppression of the MEK1/2-ERK1/2 pathway through EGFR inhibition, which has been suggested to be involved in the induction of apoptosis through the attenuation of CLDND1 expression by inhibiting ELK1 phosphorylation [15]. This indicates that CLDND1 is dependent on EGFR and that gefitinib can regulate the expression of CLDND1. Interestingly, the other claudins, CLDN1 and CLDN4, are reportedly upregulated via MEK1/2-ERK1/2 following EGF stimulation, suggesting a repressive effect of gefitinib on their expression [45]. This is consistent with the results found in this study, in which EGF-activated ELK1 phosphorylation regulated CLDND1 expression and was repressed by gefitinib. However, the effect of gefitinib on suppressing CLDND1 expression in various types of cancer cells and the kinase signaling pathway downstream of EGF signaling is yet to be elucidated, and thus is a subject for future studies.

Claudins play an important role as intercellular adhesion molecules in regulating the permeability of the intercellular barrier. However, it has also been shown that overexpression of CLDN1 has various biological disadvantages. For example, CLDN1 is not observed in a normal/healthy blood–brain barrier (BBB), but is highly expressed after stroke onset, causing leakage of the BBB [46]. Aberrant expression of claudins has been reported in a number of cancer tissues. CLDN18.2 is upregulated in advanced gastric/gastroesophageal junction and esophageal adenocarcinoma; primary EOX therapy is recommended in patients with CLDN18.2-positive cancer. Moreover, the addition of a chimeric monoclonal anti-CLDN18.2 antibody (zolvecoximab/IMAB362) to primary EOX therapy has been reported to prolong PFS and OS compared with EOX alone in these patients [13]. Furthermore, CLDND1 has been reported to be overexpressed in breast cancer cells that are basal cell-like or estrogen receptor-negative [15]. This suggests that aberrantly expressed CLDND1 can be inhibited by gefitinib treatment, in turn inhibiting the growth of these cancer cells. CLDND1 is widely distributed in the adult central nervous system with the highest expression in the corpus callosum, cerebral cortex, medulla, spinal cord, and subthalamic nucleus, and in rat optic nerves and cultured oligodendrocytes, and the myelinating cells [47]. Meanwhile, the serum CLDND1-derived peptide antibody levels are elevated in patients with cerebral infarction when compared with healthy controls [48]. The functional role of high expression levels of CLDND1 in a normal central nervous system and in cerebrovascular disease is not known, and further analysis is needed to determine the effects of gefitinib treatment on the brain tissue by suppressing CLDND1 expression.

## 5. Conclusions

This study revealed that CLDND1 is expressed in a manner dependent on ELK1 activation through EGF signaling. Moreover, our findings suggest that CLDND1 expression can be suppressed by gefitinib, a targeted molecular anticancer agent. Overall, these data demonstrate the potential of this approach as a new target in cancer therapy and vascular disease treatment.

## Data Availability

Data is contained within the article and Supplementary materials.

## Supplementary Materials

The following supporting information can be downloaded at: https://www.mdpi.com/article/10.3390/biomedicines10081792/s1, Table S1: Primers used in this study.
