# Supplementary material for: EGF-Dependent Activation of ELK1 Contributes to the Induction of CLDND1 Expression Involved in Tight Junction Formation
Source: Biomedicines. 2022 Jul 26;10(8):1792. doi: 10.3390/biomedicines10081792 (PMC9329870; doi:10.3390/biomedicines10081792)
Supplement: Supplementary file 1 [file biomedicines-10-01792-s001.zip › biomedicines-1668064-supplementary.pdf]

**Supplementary Information**

**Table S1.** Primers used in this study.

| Primer | Sequence (5′–3′) |
|---|---|
| ***Luciferase reporter and expression system cloning*** | |
| pCLDND1-FW(−742)-MluI | CGCACGCGTTCCCTGCTTCCTGTCCAT |
| pCLDND1-FW(−734)-MluI | GTCACGCGTTCCTGTCCATGAATTTCTGTG |
| pCLDND1-RV(+192)-SalI | AGCTGTCGACTCTAGCTCAGACCACAGCAC |
| PGVB2-FW | GCCCAAGCTACCATGATAAG |
| PGVB2-RV | TCATAGCTTCTGCCAACCGAAC |
| hELK1-FW-EcoRV | CCGATATCCAGACCCATCTGTGACGCTGTG |
| hELK1-RV-XhoI | GGTGCTCGAGGTAGTAGTCATGGCTTCTGG |
| hELK1-FW-del86mt-EcoRV | CCGATATCCATCCTACCCTGAGGTCGCAG |
| hELK1-FW-S383Amt | CACTTCTGGAGCACCCTGGCTCCCATTGCGCCCCGTAGC |
| hELK1-RV-S383Amt | GCTACGGGGCGCAATGGGAGCCAGGGTGCTCCAGAAGTG |
| hSRF-FW- EcoRV | CGAGATATCCATTACCGACCCAAGCTGGGG |
| hSRF-RV- XhoI | CATCTCGAGAGGGCGGCGGGCGGATCATTC |
| ***ChIP-PCR*** | |
| promoter-ChIP-FW | CCTCTGGTCCTTGAGTCAGC |
| promoter-ChIP-RV | CAGATAAGGTCATGGACAGC |
| del-ChIP-FW (PGVB2) | GCCCAAGCTACCATGATAAG |
| ***qRT-PCR*** | |
| rtCLDND1-FW | CTAACTGAGCAGTTCATGGAG |
| rtCLDND1-RV | TAAGCTTCGGCAAATGCAAG |
| rtELK1-FW | GGCCACATCATCTCCTGGAC |
| rtELK1-RV | CTTGTAGACGAACTTCTGGC |
| rtEGR1-FW | CTTCAACCCTCAGGCGGACAC |
| rtEGR1-RV | GTAACTGGTCTCCACCAGCAC |
| rt18S rRNA-FW | CGATAACGAACGAGACTCTGG |
| rt18S rRNA-RV | TAGGGTAGGCACACGCTGAGC |
